# Intraspecific Chloroplast Genome Genetic Polymorphism of *Pinellia ternata* (Xi Junecry) and Its Revelation of a Single Origin in Phylogeny

**DOI:** 10.3390/genes15121638

**Published:** 2024-12-20

**Authors:** Wenlong Xing, Weihan Yu, Yuanyuan Kong, Xian Ren, Liuying Zhu, Qingyang Li, Yujie Yang, Yueqin Cheng, Hongwei Wang

**Affiliations:** 1 College of Plant Protection, Henan Agricultural University, Zhengzhou 450002, Chinachengyq126@126.com (Y.C.); 2 Shangcheng County Bureau of Agriculture and Rural Affairs, Xinyang 464000, China; 3 Gushi County Animal Health Supervision Institute, Xinyang 464000, China

**Keywords:** *Pinellia ternata* (Xi Junecry), chloroplast genome, nucleotide polymorphisms, high variable region

## Abstract

**Background**: Xi Junecry (*Pinellia ternata*), a perennial herb of the Araceae family, is indigenous to Xinxian County, Henan Province, China, and is regarded as a premium variety among similar medicinal materials. However, the lack of comprehensive genetic information on Xi Junecry germplasm resources has constrained the cultivation and identification of high-quality varieties. **Methods**: In this study, six chloroplast genomes of Xi Junecry were assembled and annotated using high-throughput sequencing. Subsequently, comparative analyses were conducted, and a phylogenetic tree was constructed. **Results**: The six Xi Junecry chloroplast genome lengths ranged from 157,456 to 158,406 bp, and the GC content was between 36.0% and 36.2%. A total of 265 single nucleotide polymorphism sites were identified across the six genomes, with a whole-genome nucleotide diversity (Pi) value of 0.00084. Among the four genomic regions, the small single-copy region exhibited the highest Pi, followed by the large single-copy region, while the inverted repeat region showed the lowest. Nucleotide polymorphism in coding regions was significantly lower than in non-coding regions. Nine hypervariable regions were identified, as follows: *ndhE-ndhG*, *trnN-GUU-ndhF, trnS-GCU-trnG-UCC*, *atpB-rbcL*, *psaI*, *accD-ycf4*, *psbE-petL*, *psaC-ndhE*, and *psbI-trnG-UCC*. Positive selection sites were detected in the *accD* and *rbcL* genes. Phylogenetic analysis clustered the six Xi Junecry samples into a distinct clade, separating them from other regional *Pinellia* samples. **Conclusions**: These findings elucidate the genetic variation levels in Xi Junecry and provide high-variability loci for population history inference, genetic diversity assessment, species domestication studies, and new cultivar development.

## 1. Introduction

*Pinellia ternata* (Thunb.) Makino (Araceae), a perennial herbaceous plant, is a polyploid complex species with chromosome base numbers of 7, 8, 9, and 13, and ploidy levels of 2×, 4×, 6×, 8×, and 11× [1]. Xue et al. constructed the first chromosome-level genome of *P. ternata* using wild diploid samples. The genome has a size of 2.08 Gb, comprises 34,342 genes, and is organized across 13 pseudochromosomes [2]. *P. ternata* is widely recognized in traditional Chinese medicine for its dried tubers, which are pungent in taste and warm in nature. These tubers are known for their therapeutic properties, including counteracting nausea, eliminating dampness, resolving phlegm, and relieving oppression and masses [3]. Modern pharmacological studies have further demonstrated its efficacy in reducing blood lipids and blood pressure, as well as its antitumor and anti-ulcer properties [4]. The main cultivation areas for *P. ternata* include Henan, Shandong, Anhui, and Sichuan provinces in China. Among these, Xi Junecry from Xixian County, Henan, is particularly esteemed for its superior qualities, including a starch content of up to 75%. Additionally, it contains various secondary metabolites such as alkaloids, organic acids, and 17 types of amino acids. The alkaloid content ranges from 0.17% to 0.27%, while the organic acid content falls between 0.25% and 0.6% [5,6]. The best varieties are harvested along the Huaihe and Luehe Rivers, earning Xi Junecry a reputation as a geographically authentic medicinal material [7].

Traditionally, the dried tubers of *P. ternata* have been extensively used as a key ingredient in Chinese medicine, with a long history of widespread application [8]. Beyond its medicinal value, ethanol extracts from the peels of Xi Junecry have been found to inhibit the growth of aphids and other insects, suggesting its potential use as a plant-based pesticide [9]. With increasing recognition of its medicinal and agricultural value, the demand for Xi Junecry has surged significantly. This has created an urgent need for efficient germplasm identification and the development of high-quality cultivars.

Historically, the supply of Xi Junecry relied primarily on the collection of wild plants; however, industrialization, land exploitation, and the frequent occurrence of extreme weather events driven by global warming have led to the depletion of wild Xi Junecry resources [10]. The excessive use of fertilizers and pesticides, combined with overharvesting, has further degraded the quality and availability of wild populations [6,11]. Consequently, cultivated Xi Junecry has gradually become a primary source but its cultivation faces challenges due to germplasm confusion. Farmers often grow Xi Junecry alongside non-local varieties, undermining the authenticity and quality of the product [12]. As *P. ternata* primarily reproduces asexually, the lack of high-yield and high-quality cultivars has become a significant bottleneck in its artificial cultivation.

The chloroplast, a unique organelle in plant cells, has a highly conserved genome structure, gene content, and gene order. Most chloroplast genomes exhibit a typical quadripartite structure, consisting of a large single-copy region (LSC), a small single-copy region (SSC), and two inverted repeat regions (IRs) [13]. Chloroplast DNA (cpDNA) is independent of the nuclear genome and exhibits semi-autonomous replication, with maternal inheritance in most plants [14,15]. Due to their relatively stable size, structure, and gene content, chloroplast genomes are widely used for species identification, phylogenetic analysis, and studies on origin and evolution [16]. Advances in next-generation sequencing technologies have enriched the chloroplast genome database, facilitating its application in resolving phylogenetic relationships among plant taxa [17]. Moreover, the chloroplast genome encodes many genes essential for photosynthesis, and variations in the associated genetic information can affect the function of these genes, thereby influencing crop yield and traits. For instance, a specific point mutation in the 16S rRNA gene within the chloroplast genome has been shown to confer resistance to antibiotics such as streptomycin or spectinomycin [18,19]. A recent study further demonstrated that resistance to the herbicide metribuzin could be introduced into Arabidopsis thaliana through targeted base editing of the chloroplast genome [20].

In this study, we employed high-throughput sequencing technology to assemble and annotate the chloroplast genomes of six Xi Junecry individuals from its primary distribution areas. Comparative analysis and phylogenetic reconstruction were conducted to elucidate the genetic variation within Xi Junecry and its evolutionary relationship with other regional Junecry varieties. The findings provide critical insights into the genetic diversity of Xi Junecry, offering valuable DNA variation data for population history inference, cultivar development, and germplasm authentication.

## 2. Materials and Methods

### 2.1. Collection and Preservation of Materials

Fresh leaves were collected in September 2022 from six representative Xi Junecry natural populations located in Changling (CL), Guandian (GD), Pengdian (PD), Pangwan (PW), Zhangbanqiao (ZB), and Zhangtao (ZT) (Table 1). The plant materials were identified by Professor Jia-Mei Li, and voucher specimens were deposited in the Herbarium of Henan Agricultural University under the accession numbers 20220911XBX001–20220911XBX006. These populations were evenly distributed across the entire geographical range of Xi Junecry, with careful consideration given to the fact that they originated from different environmental conditions. As a result, the collected samples are representative of the genetic variation within Xi Junecry. The leaves were immediately silica-gel-dried and stored at room temperature.

### 2.2. Chloroplast Genome Assembly and Comparative Analysis of Xi Junecry

#### 2.2.1. DNA Extraction and Sequencing

Total DNA was extracted from six representative Xi Junecry samples using a modified cetyltrimethylammonium bromide (CTAB) method [21]. DNA quality and concentration were evaluated using agarose gel electrophoresis and a micro-ultraviolet spectrophotometer (Nanodrop 2000, Thermo, Shanghai, China). The extracted DNA was used to construct sequencing libraries with the NEBNext^®^ Ultra™ DNA Library Prep Kit for Illumina (NEB, Ipswich, MA, USA; Catalog #: E7370L). The library fragment size was approximately 350 bp, and paired-end sequencing (PE 150, 150 bp per end) was performed using the Illumina HiSeq 4000 platform, generating at least 6 GB of data per sample.

#### 2.2.2. Chloroplast Genome Assembly

Adapter sequences were removed from the raw reads using quality filtering software Fastp v0.19.7. During this process, 5′ non-AGCT bases were trimmed, low-quality reads with over 10% N content were discarded, and fragments shorter than 50 bp were filtered out. Chloroplast genome assembly was conducted using GetOrganelle v1.7.6.1 and NOVOPlasty v3.7.2, with the *P. ternata* chloroplast genome (MT193722) serving as the reference.

To optimize assembly results, the parameters for GetOrganelle were fine-tuned: the K-value was increased from the initial set of 45, 65, 85, and 105 to 21, 33, 45, 65, 85, 105, and 121, while the R-value was raised from 15 to 30 to enhance assembly completeness. For NOVOPlasty, the K-mer value was expanded from the initial 33 to include 33, 45, 85, and 105. Despite these efforts, the assembly did not yield a complete chloroplast genome. Consequently, the assembled fragments were aligned and evaluated for completeness and quality by mapping them to the reference genome using the “Map to Reference” tool in Geneious Prime R9. Further refinements included aligning reads to contigs and mapping to the reference genome for gap filling using GapCloser v1.12 software. Redundant sequences were removed to finalize the assembly, resulting in a more accurate chloroplast genome.

#### 2.2.3. Chloroplast Genome Annotation

The assembled chloroplast genome sequences were annotated using the CPGAVAS2 (https://link.zhihu.com/?target=http%3A//47.96.249.172%3A16019/analyzer/annotate) and GeSeq (https://chlorobox.mpimp-golm.mpg.de/geseq.html, accessed on 4 January 2023) online tools [22,23]. The annotations were aligned with the reference genome using the MAFFT tool in Geneious Prime R9, followed by manual curation. Adjustments were made to start and stop codons, exon–intron boundaries, and the names of genes and RNAs.

#### 2.2.4. Comparative Analysis of Chloroplast Genomes

Simple sequence repeats (SSRs) were detected in the six Xi Junecry chloroplast genomes using MISA software v2.1 (https://webblast.ipk-gatersleben.de/misa/). The minimum repeat numbers for mono-, di-, tri-, tetra-, penta-, and hexanucleotide motifs were set to 10, 5, 4, 3, 3, and 3, respectively [24].

Chloroplast genomes (including a single IR region) were aligned in Geneious Prime R9, and genetic diversity, including nucleotide polymorphism (Pi), was analyzed using DnaSP v6 [25]. The number of transitions and transversions among mutation sites was also calculated. Nucleotide polymorphism (Pi) values across different regions of the chloroplast genome were determined using a sliding window analysis with a window size of 600 bp and a step size of 200 bp to identify highly variable regions.

Based on the published annotations of *P. ternata* chloroplast genomes, sequence alignment was performed using the mVISTA online tool (https://genome.lbl.gov/vista/mvista/about.shtml) [26] with the Shuffle-LAGAN model to visualize sequence divergence among the six Xi Junecry samples.

#### 2.2.5. Selective Pressure Analysis

Twenty-five protein-coding sequences with nucleotide polymorphisms were extracted from Geneious Prime R9. Sequence alignment was performed using the MAFFT plugin, with manual corrections to remove stop codons. The codeml program in PAML (https://github.com/abacus-gene/paml) [27] was used to calculate the ratio of non-synonymous to synonymous substitution rates (ω = dN/dS) under site models (model = 0; NSsites = 7, 8) to infer selective pressures.

#### 2.2.6. Phylogenetic Tree Construction

The six assembled chloroplast genomes of Xi Junecry and three previously published chloroplast genomes of *P. ternata* (KR270823, MT193722, ON462056) were included as the ingroup. Two closely related species, *Arisaema erubescens* (MT676834) and *A. franchetianum* (MN046885), were used as outgroups. Genome sequences were aligned and trimmed in Geneious software. A phylogenetic tree was constructed using the ModelFinder module in PhyloSuite [28] with the maximum likelihood and Bayesian inference methods. The resulting tree was visualized and edited using FigTree v1.4.3 [29].

## 3. Results

### 3.1. Basic Characteristics of Xi Junecry Chloroplast Genomes

The raw read counts ranged from approximately 40 million in GD to 47 million in CL, with CL showing the highest read count and GD the lowest. After quality filtering, the number of clean reads slightly decreased, with CL retaining the highest count. Similarly, raw base sizes varied from 6.01 GB in GD to 7.07 GB in CL, and after cleaning, clean base sizes ranged from 5.96 GB (GD) to 7.01 GB (CL). Although the assembly of the Xi Junecry chloroplast genome in this study is incomplete, it achieved over 96% completeness compared to the reference Junecry chloroplast genome. The annotated gene types and total gene counts are highly consistent, suggesting that the six assembled Xi Junecry chloroplast genomes can adequately represent the genomic characteristics of this species for subsequent analyses (Table 2). The lengths of the six assembled chloroplast genomes ranged from 157,456 to 158,406 bp, with the shortest genome observed in the ZB individual and the longest in PW, resulting in a difference of 950 bp. The Xi Junecry chloroplast genome exhibits a typical quadripartite structure. The assembled lengths of the large single-copy (LSC), inverted repeat (IR), and small single-copy (SSC) regions were 91,363–92,345 bp, 25,174–25,250 bp, and 15,215–15,704 bp, respectively, accounting for approximately 58%, 16%, and 10% of the total genome length. GC content, an important indicator of genomic stability, was found to range between 36.0% and 36.2% across the assembled Xi Junecry chloroplast genomes.

Most genomes contained 129 annotated genes, including 85 protein-coding genes, 36 tRNA genes, and 8 rRNA genes. However, the individual ZT had 130 annotated genes, possessing an additional tRNA gene. The gene composition and arrangement were highly conserved, with 111–112 unique genes arranged in the same order. Among these, eighteen genes had two copies, including seven tRNA genes (*trnI-CAU*, *trnL-CAA*, *trnV-GAC, trnI-GAU*, *trnA-UGC*, *trnR-ACG*, and *trnN-GUU*), four rRNA genes (*rRNA4.5S*, *rRNA5S*, *rRNA16S*, and *rRNA23S*), and seven protein-coding genes (*rpl2*, *rpl23*, *ycf2*, *ndhB*, *rps7*, *rps12*, and *ycf68*) (Table 3).

### 3.2. Simple Sequence Repeats (SSRs) Analysis

**Figure 1:**
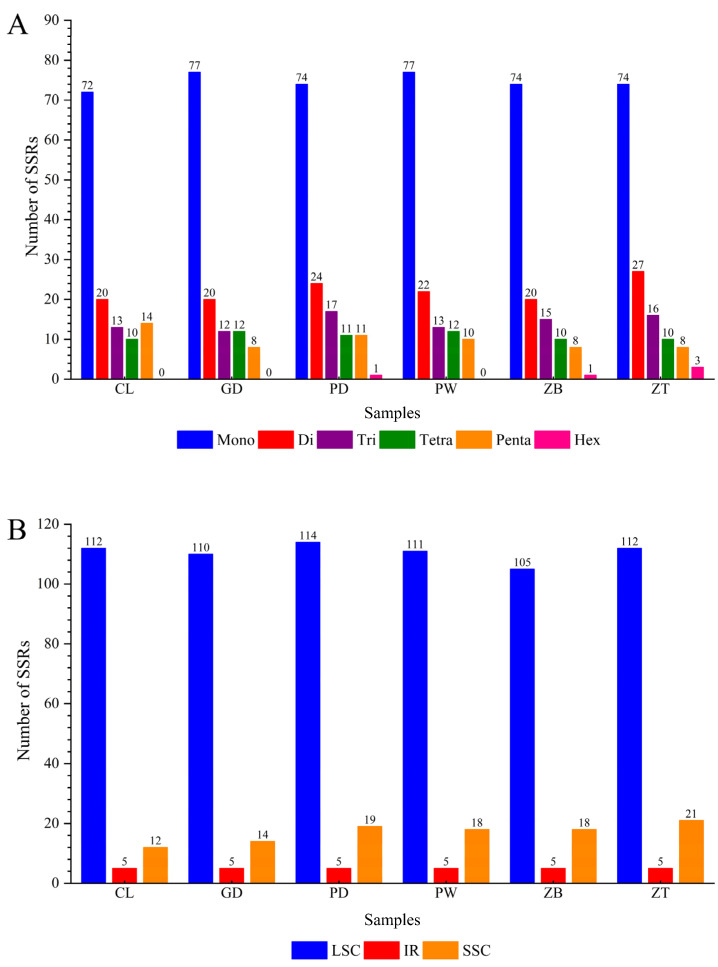
Chloroplast genome repeat analysis in Xi Junecry: (**A**) number of different types of SSR loci in the assembled green genome; (**B**) distribution of SSRs in the assembled chloroplast genome. Note: the sample names such as CL are the same as those in Table 1.

The number of SSRs detected in the six genomes ranged from 128 to 138. Mononucleotide repeats were the most abundant (72–77), followed by dinucleotide repeats (20–27), while hexanucleotide repeats were the least common (0–3). Notably, individuals CL, GD, and PW lacked hexanucleotide repeats, whereas ZT had the most hexanucleotide repeats (3) (Figure 1A). For mononucleotide repeats, the number of A/T repeats (ranging from 69 to 73) is notably higher than that of C/G repeats (ranging from 2 to 4). In dinucleotide repeat SSRs, the AT/AT type (ranging from 19 to 26) is the most prevalent. Additionally, in the trinucleotide and tetranucleotide SSRs, the most common repeat types are AAT/ATT (ranging from 10 to 15) and AAAT/ATTT (ranging from 5 to 6), respectively. Regional analysis revealed that the LSC region contained the most SSRs (105–114), followed by the SSC region (12–21), and the IR regions had the fewest (5) (Figure 1B).

### 3.3. Genetic Polymorphism Analysis

A total of 265 single nucleotide polymorphisms (SNPs) and 306 indels were detected across the six genomes. Of the 265 SNPs, only 6 had more than two nucleotide types, while the remaining 259 had just two. Transversions accounted for 198 sites (76%), outnumbering transitions, which occurred at 61 sites (24%). The most frequent base substitution was A/T (135 sites), while G/C (4 sites) was the least frequent. Non-coding regions exhibited more transversions (186) than transitions (45), while in coding regions, transitions (16) slightly exceeded transversions (12) (Table 4).

The overall nucleotide diversity (Pi) for the Xi Junecry chloroplast genome was 0.00084, and the Theta value was 0.00089. Among the four regions, the SSC region had the highest nucleotide diversity (Pi = 0.00273, Theta = 0.00301), followed by the LSC region, which exhibited about one-fourth the diversity of the SSC (Pi = 0.00075, Theta = 0.00078). The IR regions had the lowest diversity (Pi = 0.00006, Theta = 0.00005), less than one-tenth of the SSC region. Although the coding regions (62,852 bp) were slightly shorter than the non-coding regions (67,359 bp), their SNP count (28) was significantly lower than that of the non-coding regions (237). The Pi value for coding regions (0.00018) was significantly lower than that for non-coding regions (0.00146), only about one-seventh as diverse (Table 5).

**Figure 2:**
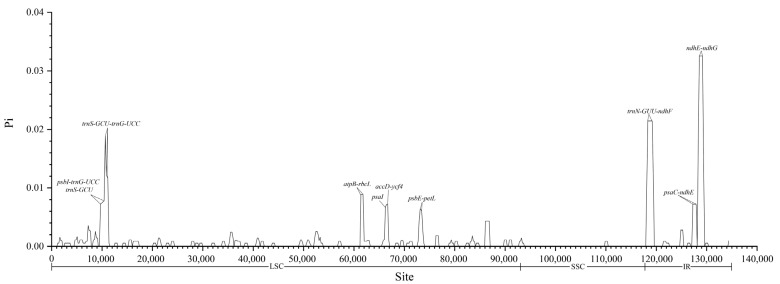
Sliding window analysis of Xi Junecry chloroplast genome.

Sliding window analysis identified nine regions with the highest nucleotide diversity: *ndhE-ndhG*, *trnN-GUU-ndhF*, *trnS-GCU-trnG-UCC*, *atpB-rbcL*, *psaI*, *accD-ycf4*, *psbE-petL*, *psaC-ndhE*, and *psbI-trnG-UCC*. Among these hotspots, three regions (*ndhE-ndhG*, *trnN-GUU-ndhF*, and *psaC-ndhE*) were located in the SSC, while the remaining six were in the LSC (Figure 2).

### 3.4. Genome Variation Analysis

**Figure 3:**
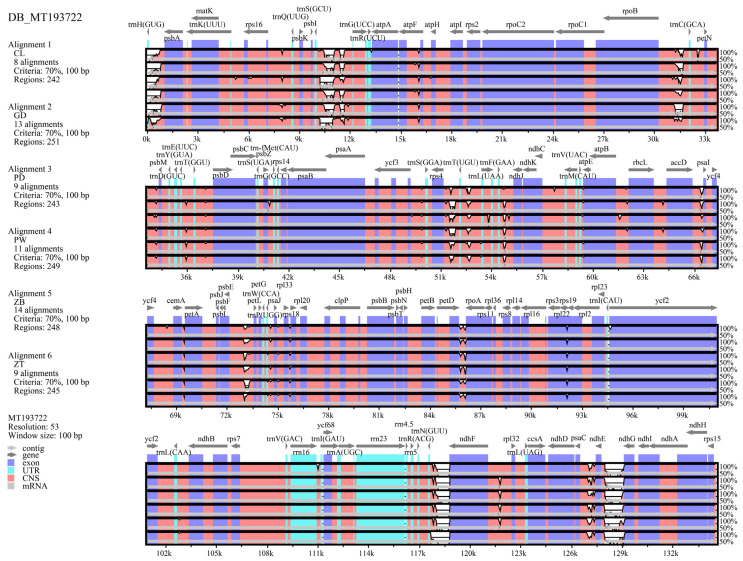
Visualization of the whole sequence alignment of six Xi Junecry chloroplast genomes.

Comparative analysis with the reference chloroplast genome (NCBI accession number: MT193722) revealed consistent structural features across the six genomes (Figure 3). Among the four regions, the LSC exhibited the highest variability, whereas the IR regions were the most conserved. Non-coding regions displayed greater variability than coding regions. However, coding regions for certain genes (*rpl22*, *atpE*, *rps18*, and *rps19*) showed distinct differences.

### 3.5. Adaptive Evolution Analysis

Selection pressure analysis using PAML identified multiple positively selected sites in the *accD* and *rbcL* genes (*p* < 0.05). The *accD* gene contained six positively selected sites (positions 42, 73, 75, 76, 77, and 79), all with posterior probabilities (PP) exceeding 0.95. In the *rbcL* gene, two sites (positions 233 and 258) were also under positive selection, with PP values greater than 0.95 (Table 6).

### 3.6. Phylogenetic Analysis

**Figure 4:**
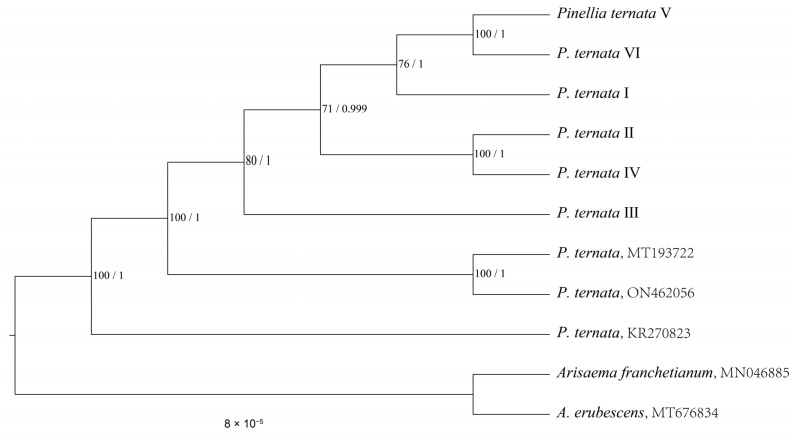
Phylogenetic tree based on whole chloroplast genome sequence (with only one inverted repeat region). Notes. *Pinellia ternate* I: CL; *P. ternate* II: GD; *P. ternate* III: PD; *P. ternate* IV: PW; *P. ternate* V: ZB; *P. ternate* VI: ZT.

To elucidate the evolutionary relationship between Xi Junecry and other *P. ternata*, phylogenetic trees were constructed using maximum likelihood (ML) and Bayesian inference (BI) methods (Figure 4). Both ML and BI analyses produced identical topologies, clustering the six Xi Junecry individuals into a distinct clade with 100 bootstraps, indicating a single origin for Xi Junecry. This clade was clearly separated from other regional *P. ternata* samples, highlighting its unique genetic lineage.

## 4. Discussion

### 4.1. The Intra-Specific Chloroplast Genome Nucleotide Mutation Patterns

In *P. ternata*, transversions (Tv) account for 76% (198 sites) of the mutations, significantly outnumbering transitions (Ts), which account for 24% (61 sites). This shows a clear bias toward transversion mutations in this species’ chloroplast genome. Non-coding regions exhibit a higher occurrence of transversions (186 sites) compared to transitions (45 sites), indicating that these regions are subject to a greater degree of mutational change. Non-coding regions typically have fewer selective constraints, potentially leading to a higher mutation rate. In coding regions, transitions (16 sites) slightly exceed transversions (12 sites), indicating a more balanced mutation distribution, likely due to the functional constraints on protein-coding genes that reduce the frequency of non-synonymous changes (which could disrupt protein function). The mutation pattern in *Scutellaria baicalensis* shows a higher number of transitions (17 sites) than transversions (8 sites), contrasting with *P. ternata*’s predominant transversion pattern. The most frequent substitution is C/T (11 times), suggesting that certain base changes (such as pyrimidine transitions) are favored in this species [30]. Similar results were found in other species. In *Stephania tetrandra*, transitions outnumber transversions, which results in a Ts/Tv ratio greater than 1 [31]. The *Leonurus cardiaca* genome shows 93 transitions and 132 transversions, with a Ts/Tv ratio of 0.705, indicating a preference for transversions. This is consistent with the pattern observed in *P. ternata*, where transversions were also more frequent [32]. The variation in mutation patterns across different species underscores the influence of selective pressures and evolutionary strategies in shaping chloroplast genome evolution. Further investigation into the underlying mechanisms could provide deeper insights into the genomic adaptation processes of *P. ternata* and related species.

### 4.2. Nucleotide Diversity of Chloroplast Genomes at the Species Level

The chloroplast genome of *P. ternata* displays a moderate nucleotide diversity (π) of 0.00084, reflecting an intermediate level of genetic variation. Compared to other species, the nucleotide diversity of *P. ternata* lies within the middle range. For example, *Euonymus maackii* exhibits a much higher nucleotide diversity (π = 0.00443), which is over five times greater than that of *P. ternata*, suggesting that *E. maackii* has a higher degree of genetic variation in its chloroplast genome [33]; on the other hand, *Larix kaempferi* has an extremely low nucleotide diversity (π = 0.00004), which is much lower than that of *P. ternata*. This suggests that *L. kaempferi* experiences a very limited amount of genetic variation in its chloroplast genome, likely due to evolutionary or ecological factors restricting genetic divergence [34].

Other species, such as *Lindera obtusiloba* (π = 0.00136) and *Chenopodium album* (π = 0.0000625), show greater and lesser genetic variation, respectively, compared to *P. ternata*. *L. obtusiloba* has a higher nucleotide diversity, indicating a relatively more diverse chloroplast genome, while *C. album* exhibits much lower diversity, pointing to a more homogeneous genetic structure within the species [35,36].

Interestingly, *Styphnolobium japonicum* (π = 0.00029) and *Bergenia scopulosa* (π = 0.00087) display genetic diversity levels that are closer to that of *P. ternata*, indicating a somewhat similar degree of genetic variation in their chloroplast genomes [37,38].

Genetic diversity is essential for the survival and sustainable utilization of species [39]. It provides the basis for adaptation to changing environmental conditions, enabling species to withstand threats such as diseases, climate change, and habitat loss [40]. High genetic variability within a population enhances resilience and reduces the risk of extinction. Furthermore, genetic diversity is critical for human development as it supports the discovery of new resources, such as medicines, crops, and industrial materials, by offering a wide range of traits for selection and innovation [41]. Conserving genetic diversity ensures long-term ecological stability and benefits both natural ecosystems and human society. Establishing a germplasm resource garden for Xi Junecry is critical for preserving its genetic diversity. As an important medicinal resource widely used in traditional medicine, Xi Junecry faces threats to its genetic diversity due to habitat loss, overharvesting, and environmental changes [6,10,11]. To protect its existing populations and prevent further genetic erosion, it is essential to establish a comprehensive germplasm repository.

### 4.3. Genetic Polymorphism Across Chloroplast Genome Regions

Genetic variation across the chloroplast genome in *P. ternata*, as observed in many species, shows significant regional differences. Among the four regions of the chloroplast genome, the IR regions exhibit the lowest genetic diversity, which is characteristic of most plant species. In *P. ternata*, the SSC region exhibits the highest nucleotide diversity, followed by the LSC region, while the IR regions remain the most conserved with the lowest diversity. This pattern aligns with findings in several other species.

For instance, in *Utricularia amethystina* [42] and *S. japonicum* [37], the IR regions demonstrate lower variability than both the LSC and SSC regions, reinforcing the generally conserved nature of the IR regions across species. Similarly, *Styrax japonicus* [43] and *Artemisia annua* [44] show that the IR regions have lower variability compared to the LSC and SSC regions, suggesting a consistent trend of conservation in the IR regions.

However, in some species, the genetic diversity in the LSC and SSC regions varies differently. For example, in *C. album* [36], the LSC region has the highest nucleotide diversity, which is over twice the diversity found in the SSC region; this contrasts with the pattern observed in *P. ternata*, where the SSC region has higher diversity than the LSC. Additionally, in *Toxicodendron vernicifluum* [45], the LSC region contains the highest number of SNPs and indels, followed by the SSC and IR regions, suggesting that some species might show higher diversity in the LSC region than the SSC, which is not typical.

In general, across all species, coding regions tend to be more conserved than non-coding regions, as seen in *P. ternata* and *S. japonicum* [37], where variations are concentrated in non-coding regions. This pattern highlights the selective constraints acting on coding regions that limit genetic variation. Furthermore, the IR regions consistently exhibit the lowest diversity as they are typically less subject to mutation due to their structural and functional roles in the chloroplast genome, which is observed in species such as *E. maackii* [33] and *Quercus acutissima* [46].

### 4.4. Highly Variable Regions of the Chloroplast Genome Within a Species

Certain regions within the chloroplast genome of *P. ternata* demonstrate marked genetic variability. The sliding window analysis identified nine hotspots of high nucleotide diversity: *ndhE-ndhG*, *trnN-GUU-ndhF*, *trnS-GCU-trnG-UCC*, *atpB-rbcL*, *psaI*, *accD-ycf4*, *psbE-petL*, *psaC-ndhE*, and *psbI-trnG-UCC*. These regions, particularly non-coding regions, show a higher mutation rate, contributing to their elevated genetic diversity. This pattern is broadly observed across multiple species, where certain regions, especially intergenic and non-coding regions, are consistently more variable.

In several species, non-coding regions are identified as key hotspots for genetic variation. For example, in *U. amethystina* [42], regions such as *trnH-psbA*, *rps16-trnQ*, and *trnC-petN* exhibit higher nucleotide diversity. Similarly, in *C. album* [36], the intergenic region *trnH-psbA* is notably variable, along with *petN-psbM* and *rpl36-infA*. In *P. ternata*, the newly added hotspots, *psbE-petL*, *psaC-ndhE*, and *psbI-trnG-UCC* further emphasize the role of non-coding regions in maintaining high genetic diversity. These regions, similar to those in other species, harbor higher SNP and InDel densities, highlighting their potential functional flexibility and evolutionary significance.

While non-coding regions generally show higher genetic diversity, some species also display considerable variation in coding regions. In *S. japonicum* [37], coding genes like *ycf1-ndhF* and *clpP* exhibit substantial diversity, with high Pi values observed in regions like *rpl36-rps8* and *ccsA-ndhD*. These coding genes are often associated with critical functions in photosynthesis and energy metabolism, which may explain their variation despite functional constraints. A similar trend is seen in *L. cardiaca* [32], where genes such as *ycf1* show marked variation, particularly with non-synonymous mutations. In *P. ternata*, although non-coding regions dominate as hotspots, coding regions like *psaI* also contribute to the overall diversity. The *psaI* gene, encoding a subunit of Photosystem I, plays a pivotal role in photosynthesis [47]. The high genetic diversity of the *psaI* gene in *P. ternata* may play a key role in its “wilting” phenomenon under stress conditions like drought, high temperature, or intense light [48]. This diversity could influence the functionality and adaptability of Photosystem I, enabling the plant to regulate light absorption and energy dissipation more effectively under stress. The wilting response may act as a survival strategy to conserve water and protect chloroplast structures by reducing photosynthetic activity. Genetic variations in *psaI* likely enhance the plant’s capacity to withstand environmental stress, suggesting an adaptive mechanism linked to its ecological resilience.

Some regions are commonly identified as hotspots of variation in multiple species. For instance, the *ycf1-ndhF* region is consistently highlighted in studies on *E. maackii* [33], *S. japonicum* [37], and *T. vernicifluum* [45], which suggests that this region might play a significant role in plant genome evolution. Likewise, the intergenic regions such as *trnH-psbA*, *psaI-ycf4*, and *trnC-petN* are repeatedly found to be highly variable across different species, reinforcing their importance in generating genetic diversity. *P. ternata* also shares some of these hotspots—particularly *psaI-ycf4*, *psbE-petL*, and *psbI-trnG-UCC*—with other species, suggesting these regions may play key roles in genome adaptation and evolution.

While there are shared hotspots, species also exhibit unique high-variation regions. For example, *Neottia listeroides* [49] identifies *trnC-trnD* and *rps15-ccsA* as hotspots, which are not as prominent in the other species discussed here. Similarly, *Q. acutissima* [46] highlights *trnG-trnR* and *rps14-psaB* as particularly variable regions, which contrasts with other species where these regions are less prominent. *P. ternata* presents a distinct set of high-variation regions, which include newly identified hotspots like *psbE-petL*, *psaC-ndhE*, and *psbI-trnG-UCC*.

Conclusion: Xi Junecry, a perennial herb of the Araceae family, is native to Xinxian County, Henan Province, China, and is highly valued for its medicinal properties. However, limited germplasm resources have hindered the cultivation and identification of high-quality varieties. This study assembled six chloroplast genomes of Xi Junecry using high-throughput sequencing, with genome sizes ranging from 157,456 to 158,406 bp and with a GC content between 36.0% and 36.2%. A total of 265 SNP sites were identified, with a whole-genome nucleotide diversity (Pi value) of 0.00084 and the SSC region exhibiting the highest diversity. Sliding window analysis revealed nine hypervariable regions, while positive selection sites were predominantly detected in the *accD* and *rbcL* genes. Phylogenetic analysis grouped Xi Junecry samples into a distinct clade, separating them from other *Pinellia* species. These findings clarify the genetic variation patterns of Xi Junecry and provide high-variability loci for population history inference, genetic diversity assessment, species domestication studies, and the development of new cultivars.

## Figures and Tables

**Table 1:** Location information of six Xi Junecry samples.

Sample Name	Sample Location	
Changling (CL)	115°6′9.9″ E	32°22′57.2″ N
Guandian (GD)	114°59′22.9″ E	32°16′15.9″ N
Pengdian (PD)	114°40′17.6″ E	32°29′54.8″ N
Pangwan (PW)	114°16′26.5″ E	32°23′56.6″ N
Zhangbanqiao (ZB)	114°53′39.6″ E	32°31′20″ N
Zhangtao (ZT)	114°52′49.7″ E	32°28′27.1″ N

**Table 2:** Structural characteristics of the chloroplast genome of six Xi Junecry.

Sample ID	Genome Length/bp	LSC/bp	IR/bp	SSC/bp	Number of Gene	Number of CDS	Number of tRNA	Number of rRNA	GC %
CL	158,318	92,345	25,174	15,625	129	85	36	8	36.0
GD	157,938	91,933	25,235	15,535	129	85	36	8	36.1
PD	157,673	91,657	25,235	15,546	129	85	36	8	36.1
PW	158,406	92,232	25,235	15,704	129	85	36	8	36.0
ZB	157,456	91,363	25,242	15,609	129	85	36	8	36.2
ZT	157,969	92,254	25,250	15,215	130	85	37	8	36.0

Notes. LSC: large single copy; SSC: small single copy; IR: inverted repeat. Sample IDs are the same as those in Table 1.

**Table 3:** List of genes in the chloroplast genome of Xi Junecry.

Category of Genes	Group of Genes	Names of Genes
Self-replication	Transfer RNA genes	*trnA-UGC* *, *trnC-GCA*, *trnD-GUC*, *trnE-UUC*, *trnF-GAA*, *trnfM-CAU*, *trnG-GCC*, *trnG-UCC*, trnH-GUG **, *trnI-CAU* *, *trnI-GAU* *, *trnK-UUU*, *trnL-UAA*, *trnL-CAA* *, *trnL-UAG*, *trnM-CAU*, *trnN-GUU* *, *trnP-GGG*, *trnQ-UUG*, *trnR-UCU*, *trnR-ACG* *, *trnS-GGA*, *trnS-UGA*, *trnS-GCU*, *trnT-GGU*, *trnT-UGU*, *trnV-UAC*, *trnV-GAC* *, *trnW-CCA*, *trnY-GUA*
	Ribosomal RNA genes	*rRNA4.5S* *, *rRNA5S* *, *rRNA16S* *, *rRNA23S* *
	Small subunit of ribosome	*rps2, rps3, rps4, rps7* *, rps8, rps11, rps12 *, rps14, rps15, rps16, rps18, rps19
	Large subunit of ribosome	*rpl2* *, rpl14, rpl16, rpl20, rpl22, rpl23 *, rpl32, rpl33, rpl36
	DNA-dependent RNA polymerase	*rpoA*, *rpoB*, *rpoC1*, *rpoC2*
Photosynthesis	Subunits of NADH-dehydrogenase	*ndhA*, *ndhB* *, *ndhC*, *ndhD*, *ndhE*, *ndhF*, *ndhG, ndhH*, *ndhI*, *ndhJ*, *ndhK*
	Photosystem I	*psaA*, *psaB*, *psaC*, *psaI*, *psaJ*
	Photosystem II	*psbA*, *psbB*, *psbC*, *psbD*, *psbE*, *psbF*, *psbH*, *psbI*, *psbJ*, *psbK*, *psbL*, *psbM*, *psbN*, *psbT*, *psbZ*
	Rubisco	*rbcL*
	cytochrome b/f complex	*petA*, *petB*, *petD*, *petG*, *petL*, *petN*
	Subunits of ATP synthase	*atpA*, *atpB*, *atpE*, *atpF*, *atpH*, *atpI*
Other genes	Envelop membrane protein	*cemA*
	Acetyl-CoA-carboxylase c-type cytochrom synthesis gene	*ccsA*
	Subunit of Acetyl-CoA-carboxylase	*accD*
	Maturase	*matK*
	Protease	*clpP*
Genes of unknown function	Conserved reading frames	*ycf2* *, *ycf3*, *ycf4, ycf68* *

Notes. Gene *: number of copies of multi-copy genes. Gene **: extra genes in ZT individuals.

**Table 4:** Statistics of different regions and different types of SNP loci in the chloroplast genome.

	Transition (S)			Transversion (V)					Other Type	Total	S/V
	A-G	T-C	Total	A-T	A-C	G-C	G-T	Total			
Whole genome	28	33	61	135	32	4	27	198	6	265	0.31
Coding region	6	10	16	3	4	1	4	12	0	28	1.33
Non-coding region	22	23	45	132	28	3	23	186	6	237	0.24

**Table 5:** Nucleotide polymorphism analysis in different regions of the chloroplast genome in Xi Junecry.

	Structural Region				Coding Region	Non-Coding Region
	LSC	IR	SSC	Total		
Total number of sites	90,344	25,174	14,827	130,345	62,852	67,359
Number of polymorphic sites	160	3	102	265	28	237
Pi values	0.00075	0.00006	0.00273	0.00084	0.00018	0.00146
Theta-W	0.00078	0.00005	0.00301	0.00089	0.0002	0.00154
Indels	228	4	74	306	15	291

Notes. LSC: large single copy; SSC: small single copy; IR: inverted repeat.

**Table 6:** Selecting genetic test results of Xi Junecry chloroplast genome.

Genes	lnL		df	*p*-Value	Positively Selected Sites		
	Null Model	Alternative Model					
*accD*	−2070.689459	−2053.870675	2	0.00000005	42	R	0.975
					73	F	0.918
					75	V	0.959
					76	S	0.986
					77	S	0.975
					79	M	0.975
*rbcL*	−2008.916522	−2002.544689	2	0.001709024	233	F	0.966
					258	M	0.967

Notes. R: Arg; F: Phe; V: Val; S: Ser; M: Met.

## Data Availability

The data that support the findings of this study are openly available in the National Center for Biotechnology Information (NCBl) (http://www.ncbi.nlm.nih.gov, accessed on 1 December 2024), under accession numbers: PQ644294-PQ644299.
